# Pi starvation-dependent regulation of ethanolamine metabolism by phosphoethanolamine phosphatase PECP1 in Arabidopsis roots

**DOI:** 10.1093/jxb/erx408

**Published:** 2017-12-22

**Authors:** Martin Tannert, Anett May, Daniela Ditfe, Sigrid Berger, Gerd Ulrich Balcke, Alain Tissier, Margret Köck

**Affiliations:** 1 Martin Luther University Halle-Wittenberg, Biocenter, Weinbergweg, Halle (Saale), Germany; 2 Institute of Plant Biochemistry, Department of Cell and Metabolic Biology, Weinberg, Halle (Saale), Germany

**Keywords:** Choline, ethanolamine, hydrophilic interaction chromatography (HILIC), PECP1, phosphatase, phosphate starvation, phosphocholine, phosphoethanolamine, phospholipid

## Abstract

A universal plant response to phosphorus deprivation is the up-regulation of a diverse array of phosphatases. As reported recently, the *AtPECP1* gene encodes a phosphatase with *in vitro* substrate specificity for phosphoethanolamine and phosphocholine. The putative substrates suggested that AtPECP1 is related to phospholipid metabolism; however, the biological function of AtPECP1 is as yet not understood. In addition, whereas lipid remodelling processes as part of the phosphorus starvation response have been extensively studied, knowledge of the polar head group metabolism and its regulation is lacking. We found that AtPECP1 is expressed in the cytosol and exerts by far its strongest activity in roots of phosphate-starved plants. We established a novel LC-MS/MS-based method for the quantitative and simultaneous measurement of the head group metabolites. The analysis of *Atpecp1* null mutants and overexpression lines revealed that phosphoethanolamine, but not phosphocholine is the substrate of AtPECP1 *in vivo*. The impact on head group metabolite levels is greatest in roots of both loss-of-function and gain-of-function transgenic lines, indicating that the biological role of AtPECP1 is mainly restricted to roots. We suggest that phosphoethanolamine hydrolysis by AtPECP1 during Pi starvation is required to down-regulate the energy-consuming biosynthesis of phosphocholine through the methylation pathway.

## Teaser

The Pi starvation-inducible *PECP1* gene encodes a phosphoethanolamine phosphatase and is required to control the biosynthesis of phosphocholine through the methylation pathway in *Arabidopsis thaliana* roots.

## Introduction

Phosphorus is an essential mineral macronutrient required for plant growth and development. The phosphate moiety is a key component of intermediates in central and energy metabolism, signalling molecules, and structural macromolecules such as nucleic acids and phospholipids. Plants respond to inadequate availability of inorganic phosphate (Pi) with tightly controlled strategies to maximize its acquisition from the rhizosphere and to improve their abilities to utilize, remobilize, and redistribute phosphate internally. These processes encompass morphological, physiological, and metabolic/biochemical adaptations which are controlled at the transcriptional and translational level and are known as the Pi starvation response (PSR) (Plaxton and Tran, 2011; Scheible and Rojas-Triana, 2015).

To cope with suboptimal soil phosphorus availability, molecular and metabolic processes are up-regulated which increase scavenging and recycling of endogenous phosphorus from phosphorus-containing molecules. Phosphatidylcholine (PtdCho) and phosphatidylethanolamine (PtdEA) are the most abundant membrane phospholipids and account for about one-third of the organically bound phosphorus in plant tissues (Poirier *et al.*, 1991; Härtel and Benning, 2000). The activation of phospholipid degradation and the substitution with lipids that do not contain phosphorus can be regarded as a salvage strategy to make more Pi available, and will result in a marked change in the lipid composition. It is characterized by an overall decrease in the phospholipid content, by replacement of phospholipids with galacto- and sulpholipids (e.g. digalactosyldiacylglycerol, DGDG; sulfoquinovosyldiacylglycerol, SQDG), and by a marked accumulation of triacylglycerol (TAG) (Misson *et al.*, 2005; Nakamura, 2013; Siebers *et al.*, 2015; Pant *et al.*, 2015*a*).

**Fig. 1.**
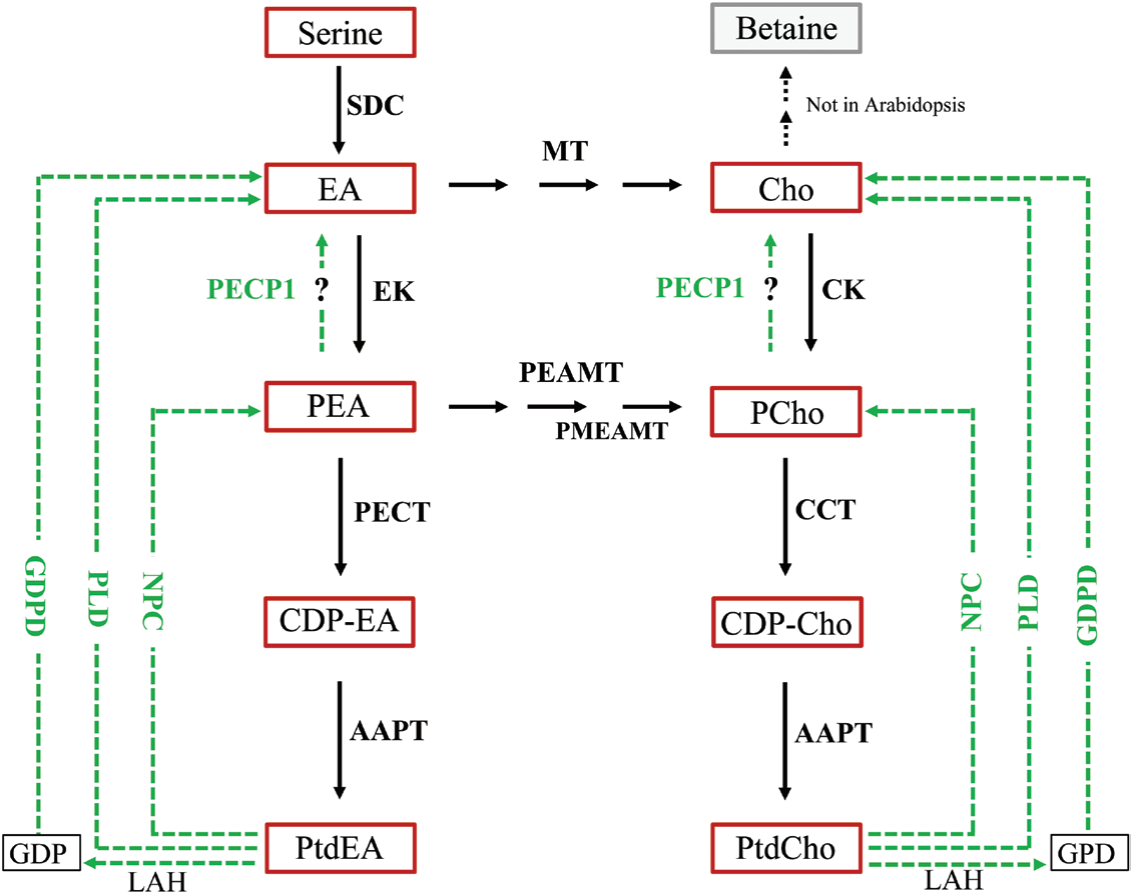
The synthesis and metabolism of ethanolamine and choline in Arabidopsis including the two branches of the Kennedy pathway. Arrows indicate *de novo* biosynthesis of phospholipids PtdEA and PtdCho, respectively. Dashed arrows highlight branches of phospholipid hydrolysis or degradation pathways that provide phosphor-base or base metabolites. Question marks indicate possible functions of AtPECP1. Compounds are framed: Cho, choline; CDP-Cho, cytidine diphosphocholine; CDP-EA, cytidine diphosphoethanolamine; EA, ethanolamine; GPD, glycerophosphodiester; PCho, phosphocholine; PEA, phosphoethanolamine; PtdCho, phosphatidylcholine; PtdEA, phosphatidylethanolamine. Enzyme names are placed on arrows: AAPT, aminoalcohol aminophosphotransferase; CCT, CTP:phosphorylcholine cytidyltransferase; CK, choline kinase; EK, ethanolamine kinase; GDPD, glycerophosphodiester phosphodiesterase; MT, metyltransferase; NPC, non-specific phospholipase C; PEAMT, phosphoethanolamine *N*-methyltransferase; PECP1, phosphoethanolamine/phosphocholine phosphatase1; PECT, CTP:phosphorylethanolamine cytidyltransferase; PLD, phospholipase D; PMEAMT, phosphomethylethanolamine *N*-methyltransferase; SDC, serine decarboxylase.

There are three different ways known to liberate Pi from phospholipids (Fig. 1). One possibility is the cleavage of phospholipids by non-specific phospholipases C (NPC4 and NPC5) which release phosphorus-containing polar head groups, primarily phosphocholine (PCho) and phosphoethanolamine (PEA), from diacyglycerol (DAG) (Nakamura *et al.*, 2005; Gaude *et al.*, 2008). A recently discovered Pi starvation-induced phosphoethanolamine/phosphocholine phosphatase, PECP1 (Fig. 1), is a first candidate for the subsequent step, the immediate liberation of Pi (May *et al.*, 2012). In a second pathway, members of the phospholipase D gene family, *PLDζ1/PLDζ2*, hydrolyse phospholipids to a free head group and phosphatidic acid (PA) (Cruz-Ramirez *et al.*, 2006; Li *et al.*, 2006). The hydrolysis of PA is carried out by phosphatidate phosphohydrolases (PAH1 and PAH2), yielding free Pi and DAG (Nakamura *et al.*, 2009; Craddock *et al.*, 2015). The third metabolic pathway is mediated by lipid acyl hydrolases (LAHs) and glycerophosphodiester phosphodiesterase (GDPD). The phospholipids are first degraded by LAHs into free fatty acids and glycerophosphodiester (GPD). GPD is further hydrolysed by GDPD into glycerol-3-phosphate (G3P) and corresponding head group moieties (Cheng *et al.*, 2011). If the original phospholipids were PtdCho and PtdEA, the head groups are choline (Cho) and ethanolamine (EA), respectively. The concentration of G3P, a metabolic link between lipid and carbohydrate metabolism, decreases strongly during Pi limitation (Pant *et al.*, 2015*b*). G3P-hydrolysing phosphatases have recently been characterized (Caparrós-Martín *et al.*, 2007). In contrast to both other routes, the third pathway results in degradation of the DAG backbone. There is evidence from recent studies that total degradation of phospholipids occurs rather than conversion into other glycolipids (Nakamura, 2013).

Pi starvation-triggered phospholipid degradation causes the release of free and phosphorylated head groups, mainly EA/PEA and Cho/PCho. Whereas lipid remodelling processes as part of the PSR have been extensively studied, knowledge of the polar head group metabolism and its regulation is lacking a deeper understanding. Xylem exudates are rich in PCho, and it has been assumed that PCho represents an important source of nutritional Pi that can be exploited by phosphorus-limited plants (Plaxton and Carswell, 1999; Misson *et al.*, 2005). A quantitative analysis of the spatial distribution of all four head group metabolites in whole plants or organs has not been published. Only few studies reported on the measurement of EA, choline, or PCho in plants (Bligny *et al.*, 1989; Gout *et al.*, 1990; Rontein *et al.*, 2003; Alatorre-Cobos *et al.*, 2012), whereas no data on PEA are available. In this context, missing experimental data hamper the understanding of the effect of Pi starvation on these metabolites and the metabolic pathways.

Plants synthesize EA from serine by serine decarboxylase (SDC; Rontein *et al.*, 2001; 2003) (Fig. 1). Choline/ethanolamine kinases (CEKs) convert free EA into PEA (Lin *et al.*, 2015). The biosynthesis of Cho requires three sequential N-methylations of an EA moiety which ultimately link EA and Cho metabolism including PtdCho biosynthesis. The methylation can occur at the levels of free EA, PEA, and PtdEA, resulting in free Cho, PCho, and PtdCho, respectively. However, the PEA-dependent pathway is regarded as the dominating methylation route, suggesting that PEA and PCho are of pivotal importance for the whole pathway (McNeil *et al.*, 2000). The reaction is catalysed by the cytosolic enzyme *S*-adenosyl-L-methionine:phosphoethanolamine *N*-methyltransferase (Bolognese and McGraw, 2000). In *Arabidopsis thaliana*, three gene loci are known which are associated with the methylation pathway (At3g18000, At1g48600, and At1g73600), but only two of them have been enzymatically characterized: At3g18000 catalyses all three methylation steps (PEAMT1; Bolognese and McGraw, 2000) and At1g48600 uses monomethylated PEA as substrate (PMEAMT; BeGora *et al.*, 2010). PtdEA and PtdCho are finally synthesized by an aminoalcoholphosphotransferase reaction which uses either CDP-EA or CDP-Cho, respectively. CDP-activated Cho and CDP-EA are synthesized by the single CTP:phosphoethanolamine cytidyltransferase (PECT) or by CTD:phosphorylcholine cytidyltransferases CCT1/2, respectively. The syntheses of the intermediates CDP-EA and CDP-Cho are considered to be the rate-limiting steps in both branches of the Kennedy pathway (Gibellini and Smith, 2010).

We recently characterized by recombinant protein technology AtPECP1, a phosphoethanolamine/phosphocholine phosphatase from *Arabidopsis thaliana* (EC 3.1.3.75) (May *et al.*, 2012). The *AtPECP1*gene has been noticed in microarray analyses as one of the most strongly up-regulated genes during Pi starvation (Misson *et al.*, 2005; Morcuende *et al.*, 2007; Müller *et al.*, 2007). Although the well-established Kennedy pathway does not comprise phosphatases, the putative substrates PEA and PCho suggested that AtPECP1 belongs to this pathway. Experimental evidence suggests that distinct enzymes of the Kennedy pathway can overlap significantly in substrate usage. For example, CEKs can use both Cho and EA as substrates, and the aminoalcoholphosphotransferase reactions are catalysed by dual-specificity enzymes capable of using both CDP-Cho and CDP-EA (Gibellini and Smith, 2010). Laboratory strains of *Chlamydomonas reinhardtii* do not contain PtdCho, but the ethanolaminephosphotransferase EPT1 possesses both ethanolamine- and cholinetransferase activities (Yang *et al.*, 2004). Several publications favour the hypothesis that plants have separate CK and EK activities, though strong experimental evidence is still missing (Tasseva *et al.*, 2004; Lin *et al.*, 2015). The question arose of whether AtPECP1 represents a dual-specificity enzyme or exhibits preference for one of the substrates.

In this study, we investigated the enzymatic and metabolic functions of Arabidopsis PECP1 in EA and Cho metabolism. To analyse the metabolic behaviour, we established a novel LC-MS/MS-based method which allows quantitative and simultaneous measurement of free and phosphorylated polar head groups. We show here that Pi starvation drastically altered metabolite levels of PEA/EA as well as PCho/Cho in an organ-dependent manner. The analysis of *Atpecp1* T-DNA insertion and ectopic overexpression lines revealed that PEA but not PCho is the substrate of AtPECP1 *in vivo*. We suggest that PEA hydrolysis by AtPECP1 during Pi starvation is required to down-regulate the energy-consuming biosynthesis of phosphocholine through the methylation pathway.

## Materials and methods

### Plant material and growth conditions

#### Hydroponic culture

Seeds of *A. thaliana* (Col-0; ABRC CS70000), T-DNA insertion lines (see below), and overexpression lines in this background were sown on wetted Grodan wool, stratified at 4 °C in the dark for 3 d, then grown hydroponically on 1/4 strength Hoagland medium for 2 weeks, followed by growth on Hoagland medium (Daram *et al.*, 1998) supplemented with 0.5 mM phosphate (+Pi) or 10 µM phosphate (–Pi) for 3 weeks with aeration under long-day conditions with 100 µmol m^−2^ s^−1^ light.

#### Axenic liquid culture of seedlings

Seedlings were grown under constant light (~40 µmol m^−2^ s^−1^) and slow shaking in media with 3 mM phosphate (full nutrition medium) or 0.2 mM phosphate (‘reduced Pi’ medium) for 7 d as described (Scheible *et al.*, 2004). To initiate Pi starvation, seedlings grown with 0.2 mM phosphate were transferred to phosphate-free conditions whereas control plants received fresh full medium. Plants were grown for an additional 2 d. To suppress Pi starvation, the Pi-starved plants received 0.5 mM phosphate.

#### Agar plate cultivation

Seeds were sterilized and sown on 0.8% agar plates containing 1% sucrose and 1/2 strength Murashige and Skoog medium without phosphate (MSP11, Caisson Laboratories, North Logan, UT, USA) or medium supplemented with Pi concentrations as indicated. As revealed by inductively coupled plasma analysis, agar (Caisson Laboratories) contains 1.7 µmol P g^−1^ agar. Seedlings were cultivated with a 16 h light (50 µmol m^−2^ s^−1^)/8 h dark cycle. To evaluate root growth, the plates were placed at a 35° angle. Plants were photographed and root lengths were determined with INKSCAPE (Open Source Scalable Vector Graphics Editor, https://inkscape.org).

### Characterization of T-DNA insertion lines

Seeds of two Arabidopsis T-DNA insertion lines (WiscDsLox341C04 and SALK_144195) were obtained from the European Arabidopsis Stock Centre (University of Nottingham). The third T-DNA mutant used (GK-350A04) was generated in the context of the GABI-Kat program (Rosso *et al.*, 2003). All lines were in the Col-0 background. Genomic DNA was isolated from leaves of ~4-week-old plants using standard procedures. PCR genotyping was performed using specific primers for the T-DNA left border and gene-specific primers corresponding to the regions flanking the respective T-DNA insertion (see primer list in Supplementary Table S1 at *JXB* online). The PCR products obtained using T-DNA primer and gene-specific primers were sequenced to confirm the location of the inserts. The knock-out status of the T-DNA mutants was analysed by real-time PCR (RT-PCR) methods using primers amplifying the coding region or primers which are placed 3' of the T-DNA insertions (Supplementary Table S1).

### Generation and analysis of *PECP1*-overexpressing lines

The transgenic lines were constructed using Gateway cloning technology. Using specific primers as listed in Supplementary Table S1, the coding sequence of *PECP1* was amplified and ligated into Entry Clone vector pENTR/D-TOPO according to the manufacturer’s instructions (Thermo Fisher Scientific). To construct the vector for ectopic expression of *PECP1*, an LR reaction was performed with the construct pENTR/D-TOPO-PECP1 and vector pGWB20 containing a 35S promoter and a C-terminal 10×Myc tag (Nakagawa *et al.*, 2007*a*). The vector construct (*PromS35:AtPECP1-cMyc*) confirmed by restriction analysis, PCR, and sequencing was transformed into *Agrobacterium tumefaciens* strain GV3101. *Arabidopsis thaliana* Col-0 plants were transformed using standard procedures (Clough and Bent, 1998). After selection of transformed plants using kanamycin, the presence of the transgene was verified by PCR with primers amplifying the complete transgene. Homozygous lines carrying single transgenic loci were isolated.

### Generation and transient expression of the eYFP construct

To construct the vector for PECP1–enhanced yellow fluorescent protein (eYFP) fusion protein expression, an LR reaction was done with the pENTR/D-TOPO-PECP1 construct and vector pGWB441 containing a 35S promoter and a C-terminal eYFP tag (Nakagawa *et al.*, 2007*b*). The confirmed construct was transformed into *A. tumefaciens* strain GV3101. Overnight-grown Agrobacteria were suspended in 5 mM MES (pH 5.5), 10 mM MgSO_4_, 150 µM acetosyringone. Leaves of greenhouse-grown *Nicotiana benthamiana* plants were infiltrated using a syringe.

### RNA extraction, cDNA synthesis, and RT-PCR

Total RNA was extracted from 100 mg samples using the GeneJET Plant RNA Purification Mini Kit (Thermo Fisher) and was treated with DNase I/RiboLock RI (Thermo Fisher) to remove genomic DNA. A test PCR was run to verify absence of residual DNA. Reverse transcription of 1 µg or 2 µg of total RNA was performed according to the manufacturer’s instructions (RevertAid H Minus reverse transcriptase, Thermo Fisher). PECP1 and UBQ primers were designed to monitor gene expression by semi-quantitative (sq)RT-PCR (see Supplementary Table S1). PCR products were separated on 1% agarose gels. Different template concentrations and a range of cycle numbers were tested for ubiquitin amplification in preliminary RT-PCR experiments to ensure application of equal amounts of total RNA.

Quantitative (q)RT-PCR was prepared in a 20 µl volume (SYBR Green PCR Master Mix, 0.2 pmol of each primer) and performed using the iCycler (Bio-Rad Laboratories). Reactions were run for 35 cycles followed by a melt curve analysis. For each qRT-PCR run, triple technical replicates were prepared and results were averaged. Relative expression data were calculated using ubiquitin expression as the reference (UBQ10; At4g05320). Levels were expressed as 40-ΔCt, except for *PECP1* expression in T-DNA insertion and overexpression lines where relative quantity (2^−ΔΔCt^) was given.

### Immunoblot analysis of PECP1 overexpression lines

To monitor tagged protein production in transgenic overexpression lines, seedlings were harvested, ground, and suspended in extraction buffer (50 mM phosphate buffer pH 7, 10 mM EDTA, 0.1% Triton X-100, 5 mM β-mercaptoethanol). Total proteins (100 µg) were separated using SDS–PAGE and transferred to a BioTrace NT nitrocellulose membrane (Pall Laboratory) [transfer buffer: 25 mM Tris, 192 mM glycine, 20% (v/v) methanol, 0.1% (w/v) SDS]. Immunodetection was performed using standard procedures. A 1:1000 dilution of mouse anti-human c-Myc antibody (MoBiTec GmbH, Germany) was applied, followed by incubation with a secondary antibody [1:40000 dilution, goat anti-mouse horseradish peroxidase (HRP) conjugated, Sigma]. The membrane was developed using Pierce ECL Western Blotting Substrate according to the instructions of the manufacturer (Pierce/Thermo Fisher).

### Measurement of enzymatic activity of PECP1

Plant material was ground in liquid N_2_. Samples (200 mg) were suspended in 600 µl of extraction buffer (50 mM HEPES pH 7.5, 50 mM MgCl_2_, 10 mM EDTA, 2 mM DTT, Sigma Protease-Mix), vortexed, centrifuged, and supernatants kept for further analyses. The enzyme activity assay (1.5 ml) containing 50 mM HEPES pH 7.5, 10 mM MgCl_2_, substrate (15 mM PEA or 5 mM PCho) was started by adding 300 µl of supernatant and incubated for up to 60 min. After several intervals, the reaction was stopped by mixing 0.3 ml of the assay mixture with 0.3 ml of 20% trichloroacetic acid and centrifuged. Supernatants (0.5 ml) were assayed for phosphate by adding equal volumes of reagent I [40 mM (NH_4_)_6_Mo_7_O_24_, 2.5 N H_2_SO_4_] and reagent II (21 mM NH_4_VO_3_, 0.28 N HNO_3_). The absorption was measured at 405 nm. The amount of phosphate generated was calculated using a calibration curve. Protein concentration of the extract was determined by Bradford assay (Carl Roth).

### Measurement of inorganic phosphate

Fresh tissues (150 mg) were homogenized in 1 ml of 10% trichloroacetic acid, left on ice for 30 min, and centrifuged at 15000 *g* for 20 min (Rebeille *et al.*, 1982). Supernatants were diluted and 0.5 ml aliquots were used in the ammonium molybdate method as stated for AtPECP1 enzyme activity measurement.

### Extraction and measurement of head group metabolites

#### Extraction

To extract polar metabolites, 250–500 mg of fresh plant material was homogenized under liquid nitrogen in a mortar and freeze-dried. A 15 mg aliquot of freeze-dried tissue was treated with 900 µl of dichloromethane/acetone (3:1, –80 °C) and 200 µl of 50 mM ammonium formate (pH 3, 4 °C) containing all four internal standards (20 mg l^–1^ PCho-d9, 10 mg l^–1^ PEA-d4, 20 mg l^–1^ Cho-d9, and 10 mg l^–1^ EA-d4). The extraction was performed in 1.6 ml cryo-tubes (Precellys Steel Kit 2, 8 mm, Peqlab, VWR) using a bead mill (FastPrep24 instrument, MP Biomedicals) with acceleration of 5.5 m s^–2^ for 60 s. After centrifugation at 13 000 rpm (5 °C, 5 min), the upper phase was transferred into a 1.5 ml tube and kept on ice. Subsequently, a second extraction was executed under the same conditions using 150 µl of 50 mM ammonium formate (pH 3) with all four internal standards. The upper phases were combined, and 20% (v/v) acetonitrile was added. A 10 µl aliquot of the solution were injected in the LC-MS/MS system for analysis.

#### Hydrophilic interaction chromatography-coupled MS (HILIC-MS/MS)

All analyses were performed with a UPLC System (ACQUITY UPLC, Waters) coupled with a QTrap^®^ 6500 mass analyser (SCIEX). For HILIC analysis, a Nucleoshell HILIC column, 2.7 µm (2.0 mm×150 mm) (Macherey-Nagel) was used at a temperature of 40 °C. The flow rate was 0.4 µl min^–1^ using a gradient of 20 mM ammonium formate pH 2.5 (solvent A) and 90% acetonitrile/10% 20 mM ammonium formate pH 2.5 (solvent B). The run time of the gradient was set to 20 min with the following profile: 0–5 min isocratic 100% B, 5–12 min 100–30% B, 12–14 min isocratic 30% B, 14–16 min 30– 100% B, 16–20 min isocratic 100% B. MS/MS detection was done by electrospray ionization (ESI)-MS/MS, operating in positive and negative ion mode. The ESI source operation parameters for positive mode were as follows: curtain gas, 40 psi; ion spray voltage, 5500 V; temperature, 450 °C; gas1, 60 psi; gas2, 70 psi; EP, 10 V. For negative mode, the following parameters were used: curtain gas 40 psi; ion spray voltage –4500 V; temperature, 450 °C; gas1, 60 psi; gas2 ,70 psi; EP, –10 V. The detection of metabolites was carried out by multiple reaction monitoring (MRM). Compound-dependent parameters for authentic standards were optimized individually by flow injection experiments (Supplementary Table S2). The evaluation of the data obtained was performed with PeakView and MultiQuant (SCIEX).

### Lipid extraction, separation, quantification, and data analyses

Root samples (300 mg) homogenized under liquid nitrogen were sent to and analysed by metaSysX GmbH using metaSysX standard procedures and in-house software (metaSysX GmbH, Potsdam-Golm, Germany). All lipids were grouped into their lipid classes, summed, and normalized to 1/10 median of all lipid classes. Three biological replicates with three plants each were analysed.

### Statistical analysis

Data sets were statistically analysed by one-way or two-way ANOVA followed by the Holm–Sidak post-hoc test for testing differences between genotypes, organs, and phosphate treatments. Different letters were used to indicate means that differ significantly (*P*<0.05).

## Results

### Regulation of *AtPECP1* gene expression by Pi starvation

**Fig. 2.**
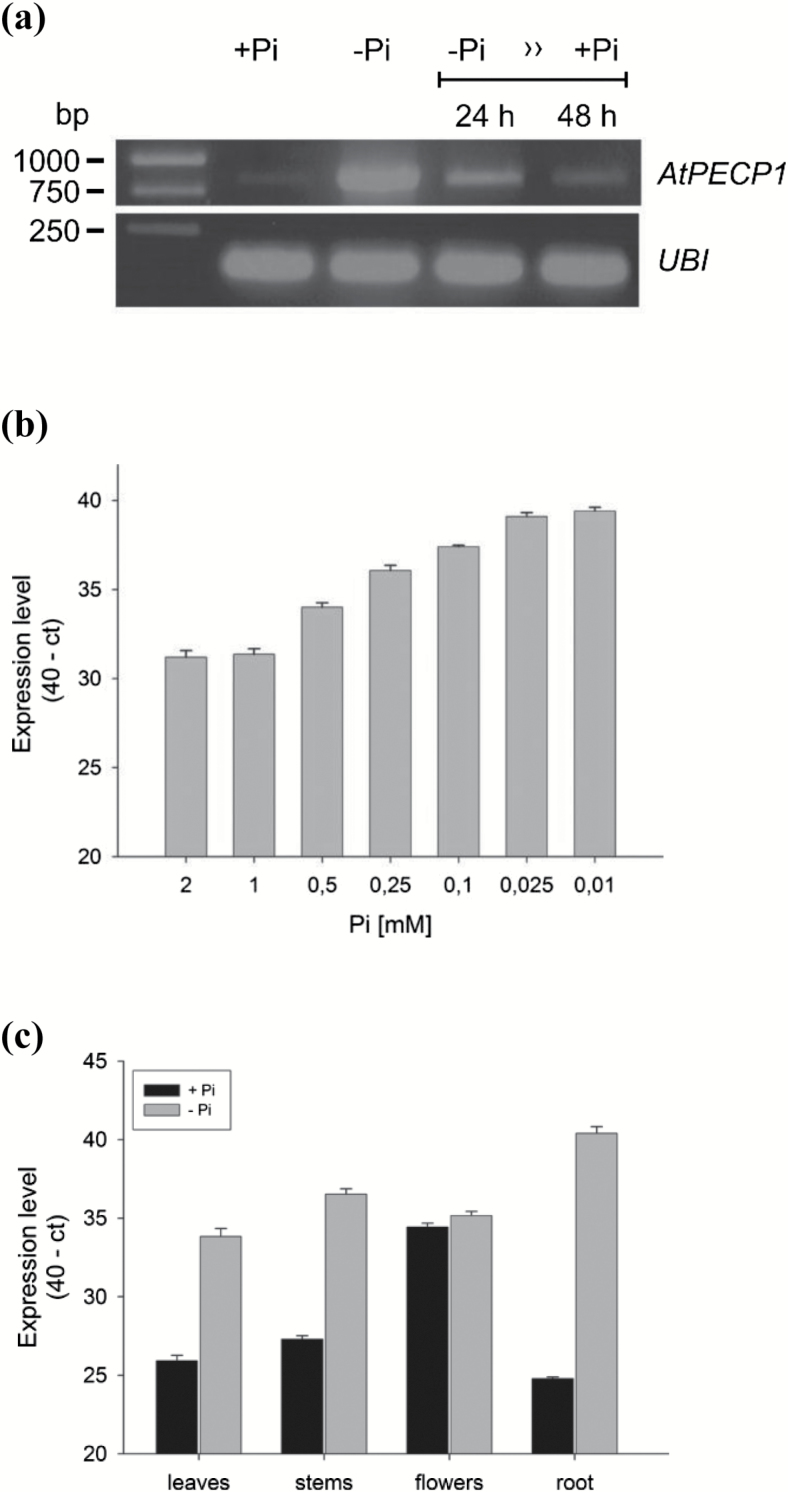
Analysis of *AtPECP1* expression in response to Pi supply. (a) Plants were grown in liquid culture. *AtPECP1* transcript levels and those of the internal control *UBQ10* were determined by RT-PCR. Transcripts were amplified for 30 cycles. Lane +Pi, plants were grown for 7 d+2 d in full nutrition medium; lane –Pi, plants grown for 7 d in ‘reduced Pi’ medium were transferred to Pi-free conditions for 2 d; lanes 24 h/48 h, –Pi plants received 0.5 mM phosphate and grew for an additional 24 h or 48 h, respectively. (b) qRT-PCR analysis of *AtPECP1* in 14-day-old Arabidopsis seedlings grown in the presence of decreasing Pi concentrations in the solid agar medium and under long-day conditions. (c) qRT-PCR analysis of *AtPECP1* expression in organs of 35-day-old plants cultivated hydroponically. The expression levels in (b) and (c) are expressed as 40-ΔC_t_. ΔC_t_ is the difference in qRT-PCR threshold cycle number between *AtPECP1* and the reference gene *UBQ10*. Expression levels shown are the mean ±SD from two biological replicates with three technical replicates for each.

Our analyses show that *AtPECP1* is tightly regulated by Pi supply. *AtPECP1* transcripts were only scarcely detectable under normal growth conditions (Fig. 2a, lane +Pi). The transcript level increased strongly after onset of Pi deprivation (Fig. 2a, lane –Pi). Additionally, we tested whether the transcriptional response can be reversed. To suppress Pi starvation, the Pi-starved plants received 0.5 mM phosphate. A marked decrease of steady-state transcript amounts was detected after 24 h and reached close to basal levels after 48 h, suggesting a stringent response of *AtPECP1* to Pi shortage (Fig. 2a). Next, to find out at which Pi concentration transcriptional activation of *AtPECP1* is initiated, we grew seedlings on agar plates supplemented with a decreasing Pi level for 14 d. We recognized a concentration-dependent increase of *AtPECP1* transcript amounts (Fig. 2b). At 0.5 mM Pi in the medium, the expression level was ~6-fold greater compared with 1 mM Pi supply, whereas growth in the presence of 0.01 mM Pi resulted in a 260-fold greater transcript abundance. To analyse the spatial *AtPECP1* expression, Arabidopsis plants were cultivated in a hydroponic system and organ samples were collected from 35-day-old adult plants. Under Pi-replete growth conditions, we found the greatest and lowest *AtPECP1* transcript levels in flowers and roots, respectively (Fig. 2c). We also tested the relative distribution of *AtPECP1* transcript amounts in soil-grown plants. Again, the greatest transcript level was observed in flowers in comparison with other organs, illustrating that expression profiles do not depend on cultivation conditions (Supplementary Fig. S1a). Plants grown hydroponically under Pi-limiting conditions (10 µM phosphate) showed typical Pi starvation symptoms such as growth retardation and anthocyanin accumulation, but flower development was unaffected (not shown). Growth under phosphate-depleted conditions resulted in 240-, 600-, and ~50000-fold greater transcript abundance for *AtPECP1* in leaves, stems, and roots, respectively, compared with organs of phosphate-replete plants (Fig. 2c). The transcript levels in flowers of both phosphate-replete and phosphate-starved plants were similar (Fig. 2c). We analysed the subcellular expression of AtPECP1 *in planta* as a fusion with eYFP and found a cytoplasmic localization in *N. benthamiana* cells (Supplementary Fig. S1b). The subcellular localization is in accordance with the predicted absence of sorting signals in the primary sequence as stated by May *et al.* (2012).

### Pi starvation drastically reduces phosphoethanolamine and phosphocholine levels in roots

**Fig. 3.**
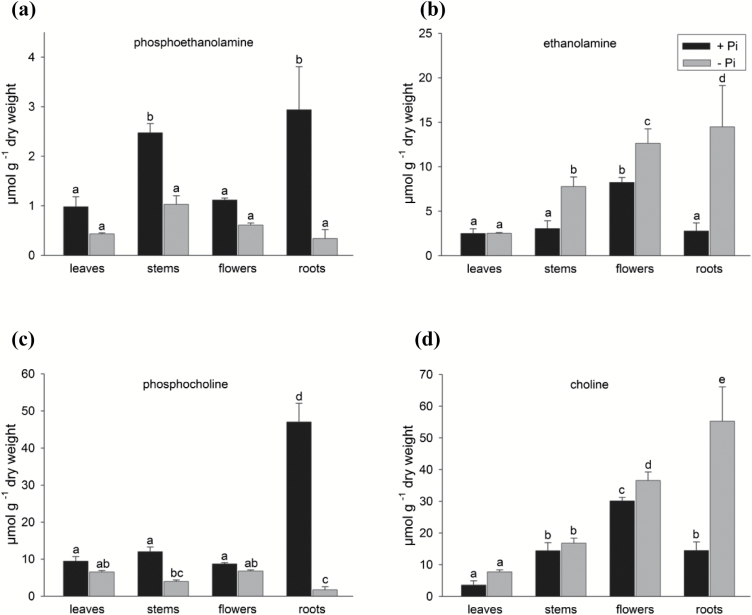
Pi starvation changes the content of head group metabolites in plant organs of wild-type plants. Content of the head group metabolites phosphoethanolamine (a), ethanolamine (b), phosphocholine (c), and choline (d) in different organs of mature Arabidopsis plants determined using HILIC-MS/MS. The key as seen in (b) applies to (a)–(d). Samples were taken from plants grown hydroponically on +Pi medium (0.5 mM) or –P medium (10 µM) under long-day conditions for 35 d. Values are the mean ±SD of measurements on at least three biological samples with several technical replicates. Two-way ANOVA was used to evaluate the differences between treatments and organs. Different letters indicate means that differ significantly (*P*<0.05).

AtPECP1 was recently characterized by recombinant protein technology as a phosphatase of the HAD (haloacid dehalogenase) superfamily with substrate specificity for PEA and PCho (May *et al.*, 2012). EA, PEA, Cho, and PCho were determined by using HILIC-MS/MS. In general, all organs of Pi-replete plants contained low concentrations of PEA and EA, ranging from 1 µmol g^−1^ to 3 µmol g^−1^ based on DW (Fig. 3). A remarkable exception was flowers, where a concentration of 8 µmol EA g^−1^ DW was found. Flowers also contained a greater Cho content at ~30 µmol g^−1^ DW, twice as much as that of roots and stems and >8-fold greater than in leaves (3.5 µmol g^−1^ DW). Intriguingly, a root PCho concentration of ~47 µmol g^−1^ DW represented by far the greatest value of all measurements and was also 4- to 5-fold greater than in leaves, flowers, or stems. Published data on PCho and Cho contents in Arabidopsis are hardly comparable because different developmental stages were used (seedlings and mature plants) or data were expressed on a fresh or dry weight basis. However, notably, the partitioning of PCho and Cho is in general agreement with what was previously observed in young seedlings where the root PCho content exceeded the shoot PCho level, and the root PCho content was much greater than the root Cho content (Alatorre-Cobos *et al.*, 2012).

Phosphate deficiency affects the steady-state levels of many phosphorylated metabolites and results in hydrolysis or degradation of phosphate-containing molecules (Plaxton and Tran, 2011). In phosphate-starved plants, we determined a decrease of phosphorylated metabolites (PEA and PCho) in all organs which was accompanied by an increase of EA and Cho levels (Fig. 3a–d). By far the most substantial change was observed in roots where the PEA and PCho levels dropped to 12% and 3.5% of the concentration in P-replete plants, respectively (Fig. 3a, c). In Pi-starved roots, EA and Cho levels were increased to 5-fold and 4-fold, respectively (Fig. 3b, d).

### PECP enzyme activity and head group metabolite levels in Pi-replete and Pi-starved knock-out Arabidopsis plants

**Fig. 4.**
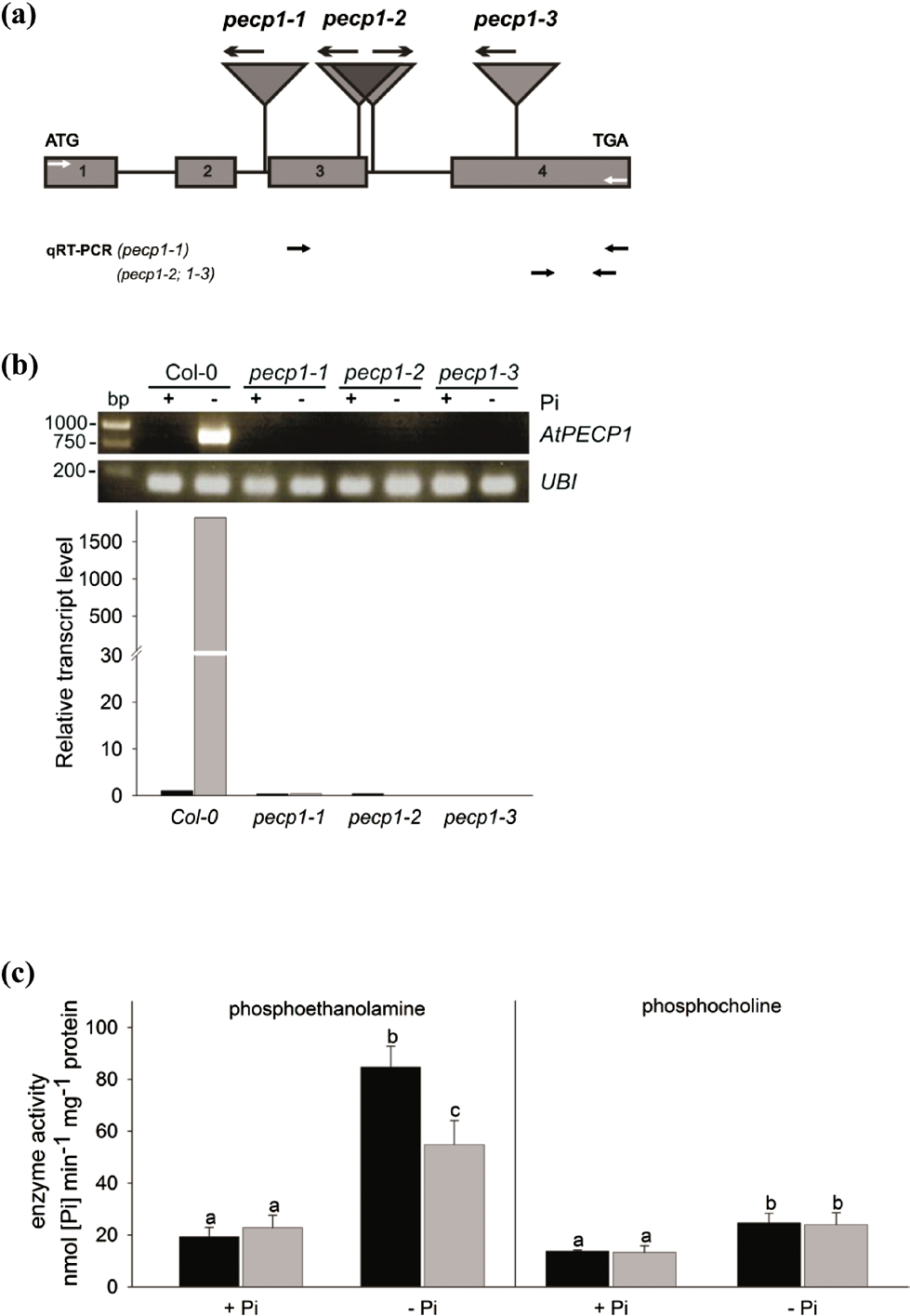
Molecular analysis of three independent *PECP1* Arabidopsis knock-out mutant alleles. (a) Positions of T-DNA insertion in the *AtPECP1* gene. Arrows (above triangles) mark the orientation of the T-DNA-derived primers (see Supplementary Fig. S2 for details of PCR genotyping and Supplementary Table S1 for primers used). Grey arrows (in boxes 1 and 4) mark positions of primers used for sqRT-PCR (amplification of the coding sequence, 840 bp). Primer positions used for qRT-PCR are shown below the gene model (all arrows not to scale). (b) RT-PCR analyses of *PECP1* expression under Pi-replete (+Pi) and Pi starvation (–Pi) conditions revealed the presence of *pecp1* null alleles. Upper image: agarose gel separation shows sqRT-PCR amplification products. Note: in contrast to Col-0, no amplification products were observed, either in P-replete or in P-starved *pecp1* seedlings (loading control UBQ10). Diagram below: qRT-PCR with primers placed 3' of T-DNA insertions as shown in (a). Calibrator sample: +Pi/Col-0; normalized to *UBQ10*. (c) Measurement of PECP1 enzyme activity with substrate phosphoethanolamine or phosphocholine, respectively, in the *pecp1-1* mutant line compared with Col-0. Samples were harvested from liquid cultured seedlings grown in +Pi or in Pi-free medium for 2 d (–Pi). Two-way ANOVA was used to evaluate the differences between genotypes and treatments. Values are means ±SD (*n*=3–5 biological replicates). Different letters indicate means that differ significantly (*P*<0.05).

To gain insight into the function of the Pi starvation-induced gene *PECP1* in Arabidopsis, three allelic T-DNA insertion lines (*pecp1-1*, *pecp1-2*, and *pecp1-3*) were identified from independent collections (Fig. 4a). The T-DNA insertion in *pecp1-1* (GK-350A04) was found to be located in the second intron (461 nucleotides downstream of the ATG codon). In *pecp1-2* (WiscDsLox341C04), two linked T-DNAs were inserted at the end of the third exon and at the beginning of the third intron (639/676 nucleotides downstream), whereas in *pecp1-3* (SALK_144195), the T-DNA was found to be inserted at nucleotide position 977 (fourth exon). Plants homozygous for the respective T-DNA insertion were directly selected from provided seeds or generated by self-pollination (Supplementary Fig. S2 shows PCR genotyping of T-DNA insertion lines). Both sqRT-PCR (amplification of the coding region, 840 bp) and qRT-PCR analyses of all T-DNA insertion lines (primers were placed 3' of insertions; Fig. 4a) showed that transcript amounts in mutants were below the limit of detection (Fig. 4b). Most importantly, the strong increase of *PECP1* transcript abundance observed in Pi-starved Col-0 seedlings was absent in Pi-starved *pecp1* mutants, indicating complete knock-outs. Examination of all homozygous *pecp1* alleles revealed no obvious phenotypic abnormalities.

We determined to what extent enzyme activity was compromised in the *pecp1* knock-out lines. Growth under Pi-replete conditions did not alter enzyme activity as measured by hydrolysis of PEA or PCho in protein extracts of *pecp1* mutant lines in comparison with those from Col-0 wild-type plants, shown here for *pecp1-1* (Fig. 4c). When grown in liquid culture with Pi-depleted medium followed by growth without Pi for 2 d, the extractable PEA-hydrolysing phosphatase activity increased in wild-type plants (4.4-fold) but the increase of activity was significantly reduced in *pecp1-1* (2.4-fold) (Fig. 4c). Using PCho as substrate, a Pi starvation-induced increase of enzyme activity was also detected (1.8-fold) but there was no difference between the wild type and the *pecp1-1* knock-out line.

**Fig. 5.**
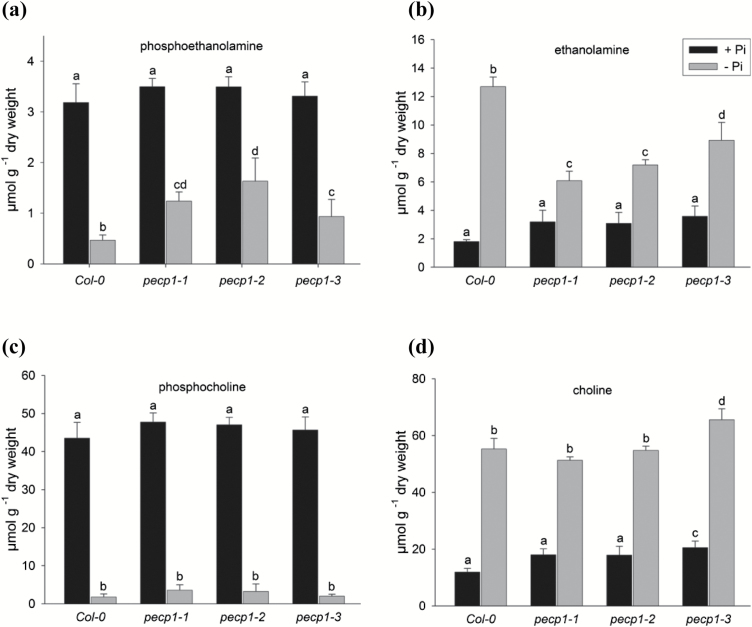
Loss of PECP1 activity extenuates Pi starvation-triggered change of PEA/EA levels. Content of head group metabolites in the roots of *pecp1* knock-out lines compared with the Col-0 wild-type control determined using HILIC-MS/MS: (a) phosphoethanolamine, (b) ethanolamine, (c) phosphocholine, and (d) choline. The key as seen in (b) applies to (a)–(d). Samples were taken from plants grown hydroponically on +Pi medium (0.5 mM) or –P medium (10 µM) under long-day conditions for 35 d. Two-way ANOVA was used to evaluate the differences between genotypes and treatments. Values shown represent the mean ±SD (*n*=5 biological replicates). Different letters indicate means that differ significantly (*P*<0.05).

Next, the levels of head group metabolites were determined in roots of Pi-replete or Pi-starved plants to test for the impact of *pecp1* knock-out on metabolite levels (Fig. 5a–d). While there was no significant change in PEA levels between Col-0 and the *pecp1* mutant lines under Pi-replete conditions, the Pi starvation-induced decrease of PEA level in Col-0 roots by 85% was significantly attenuated in *pecp1* mutants (Fig. 5a). In Pi-replete Col-0 plants and all mutant lines, EA levels were similar. While Pi starvation resulted in a 6-fold increase of the EA level in wild-type roots, the accumulation of EA in *pecp1* lines was significantly smaller (Fig. 5b). A comparison between Pi-replete wild-type and mutant plants revealed no significant differences in PCho and Cho levels. Moreover, the drastic decrease of the PCho level as well as the increase in Cho level in roots of wild-type plants (see Fig. 3c, d) were also observed in Pi-starved *pecp1* lines (Fig. 5c, d). We measured free Pi concentrations in 14-day-old Col-0 and *pecp1* seedlings as well as in shoots and roots of 28-day-old Col-0 and *pecp1* mutant plants cultivated under phosphate-replete or phosphate-depleted conditions. The disruption of *AtPECP1* gene function did not cause significant changes in free Pi concentrations (Supplementary Fig. 3).

### Loss of PECP1 activity exacerbates biochemical and morphological effects of Pi starvation

In order to investigate the impact on phospholipid catabolism in roots during Pi starvation we quantified the lipid composition in 35-day-old wild-type Col-0 plants and *pecp1-1* plants grown hydroponically in either sufficient (0.5 mM) or limiting (10 µM) Pi conditions.

In roots of Col-0 plants cultivated under Pi-depleted conditions, relative amounts of PtdEA and PtdCho decreased by 30% and 23%, respectively (Table 1). In contrast, an increase in the relative amount of DGDG (5-fold) was observed under Pi-depleted conditions. Similarly, the relative amount of sulpholipids (SQDG), another class of non-phosphorus lipids, increased 4-fold. We also found elevated relative amounts of TAGs in Pi-starved Col-0 roots (2-fold). Pi-starvation-dependent alterations of the relative amounts of lipid classes as shown here are in line with data from other studies which analysed roots and shoots independently (Cruz-Ramirez *et al.*, 2006, Pant *et al.*, 2015*a*).

**Table 1.** Loss of AtPECP1 activity exacerbates phospholipid degradation upon Pi starvation

	+Pi		–Pi	
	Col-0	*Atpecp1-1*	Col-0	*Atpecp1-1*
PtdEA	71.60 ± 12.49 a	64.03 ± 3.39 a	50.69 ± 4.55 b	40.07 ± 4.96 c
PtdCho	265.81 ± 31.50 a	245.58 ± 15.41 a	204.00 ± 14.99 b	162.13 ± 16.18 c
SQDG	0.43 ± 0.16 a	0.37 ± 0.08 a	1.79 ± 0.08 b	2.25 ± 0.38 c
MGDG	8.95 ± 0.69 a	8.23 ± 0.53 a	12.09 ± 0.36 b	11.18 ± 0.56 b
DGDG	2.87 ± 0.23 a	2.72 ± 0.08 a	14.97 ± 0.12 b	15.79 ± 0.70 b
DAG	8.81 ± 4.02 a	8.69 ± 3.83 a	7.13 ± 4.08 a	6.29 ± 3.19 a
TAG	13.77 ± 0.77 a	14.03 ± 0.88 a	30.09 ± 2.63 b	31.93 ± 4.56 b
LysoPE	0.68 ± 0.13 a	0.67 ± 0.02 a	0.32 ± 0.01 b	0.32 ± 0.07 b
LysoPC	0.78 ± 0.23 a	0.77 ± 0.05 a	0.32 ± 0.03 b	0.34 ± 0.06 b

Comparison of root lipid composition between 35-day-old wild-type plants (Col-0) and T-DNA-tagged AtPECP1 mutant plants (*Atpecp1-1*) during Pi-replete and Pi-starved growth. Data shown are relative and represent normalized intensity units. Two-way ANOVA was used to evaluate the differences between genotypes and treatments. Values are means ±SD (*n*=3 biological replicates with three plants each). Different letters indicate means that differ significantly (*P*<0.05).

In the *pecp1-1* mutant grown under Pi-replete conditions, the relative amounts of all lipid classes were similar to those in Col-0 roots. However, we observed a reduction of the relative amounts of PtdEA and PtdCho by 20% in Pi-starved *pecp1-1* roots compared with Pi-starved Col-0 roots, while relative amounts of SQDG were elevated by 25%. These results suggest that the loss of AtPECP1 activity enhances biochemical symptoms of the nutritional stress response by increasing phospholipid catabolism.

**Fig. 6.**
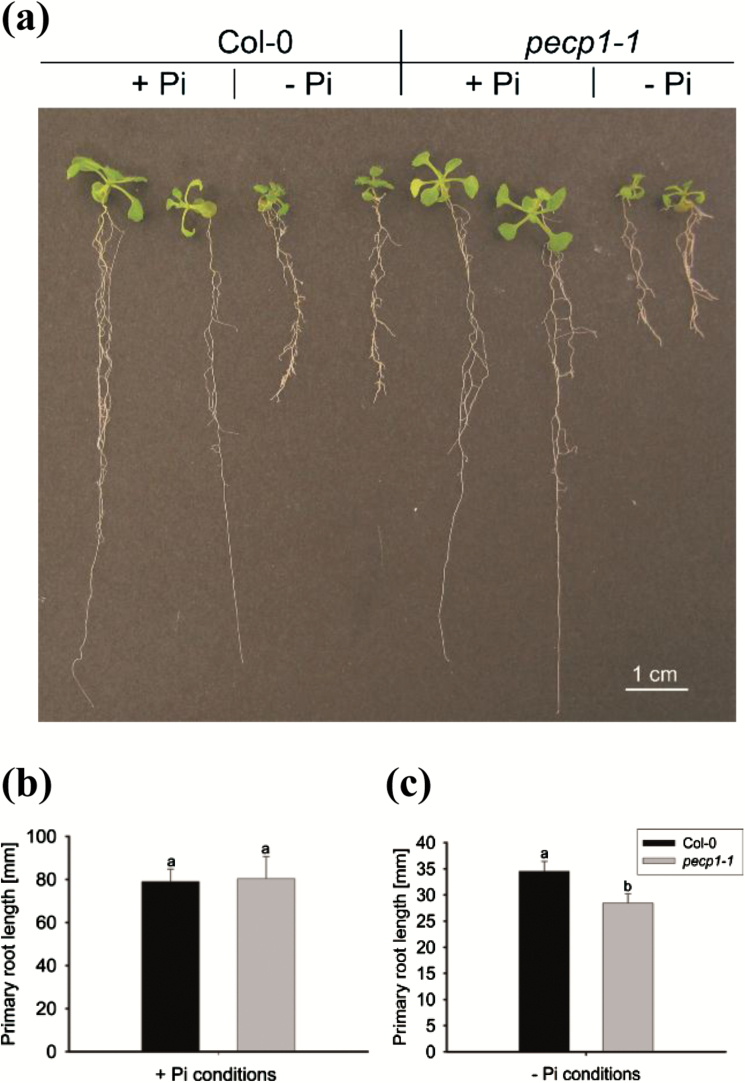
Pi starvation-inhibited root growth is exacerbated in *PECP1* knock-out plants. (a) Phenotype of 12-day-old *pecp1-1* plants compared with Col-0 plants, grown under Pi-replete (+Pi) conditions or under Pi starvation (–Pi). (b) Primary root lengths of Pi-replete Col-0 and *pecp1-1* seedlings; same key as seen in (c). (c) Primary root lengths of P-starved Col-0 and *pecp1-1* seedlings. One-way ANOVA was used to evaluate the differences between genotypes. Values represent the mean ±SE (*n*=6 in Pi-replete conditions; *n*=16–19 in Pi-starved conditions). Different letters indicate means that differ significantly (*P*<0.05).

Although *AtPECP1* expression is suppressed in the mutant lines under Pi-replete growth conditions, we observed no obvious phenotypic differences in growth or development between wild-type and *Atpecp1* plant lines. Because *PECP1* is predominantly expressed in Pi-starved roots, we investigated the potential effect of the *PECP1* insertional mutation on roots. A well-known Pi starvation-dependent developmental stress response of Arabidopsis is the suppression of primary root elongation as well as the modification of root system architecture (Williamson *et al.*, 2001). Wild-type seedlings grown under Pi-depleted conditions have a shorter primary root and more lateral roots than Pi-replete plants (Fig. 6a), as observed in earlier studies (López-Bucio *et al.*, 2002). The suppression of primary root growth was even more pronounced in *pecp1-1* mutant plants that had shorter primary roots than Col-0 (Fig. 6c).

### Constitutive ectopic expression of PECP1 reduces PEA and PCho levels

**Fig. 7.**
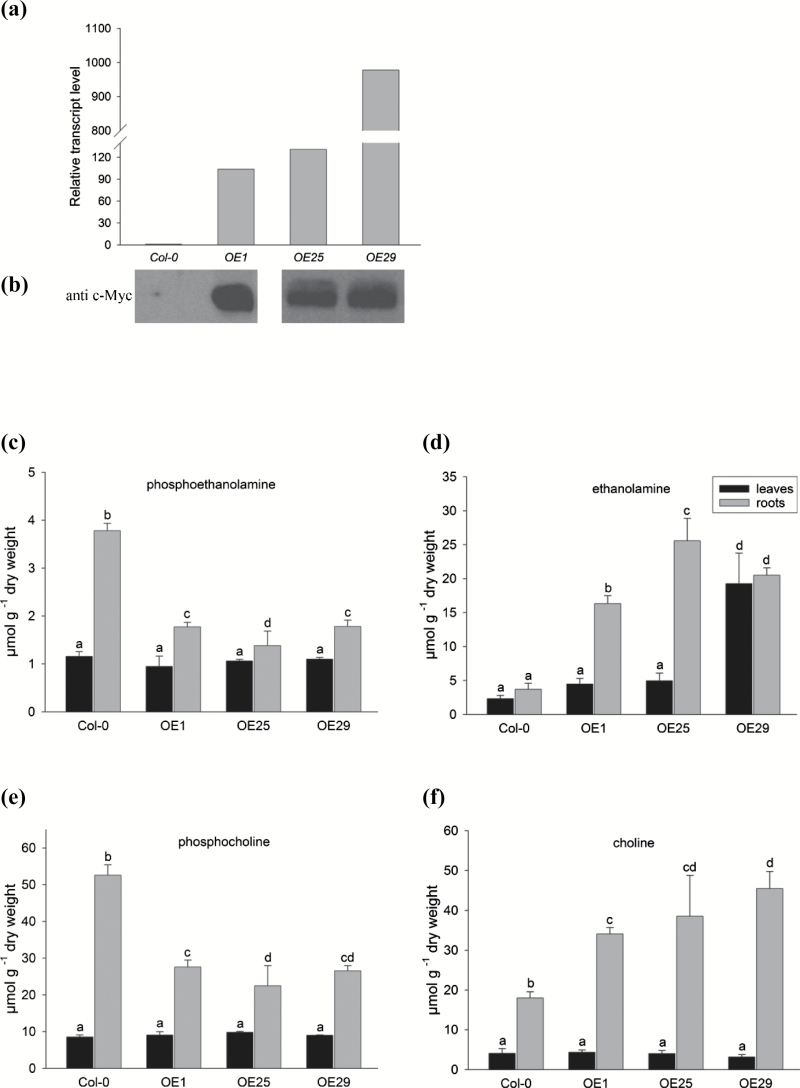
Characterization of the ectopic overexpression lines (*PromS35:AtPECP1-cMyc*). (a) qRT-PCR analysis of *AtPECP1* expression in seedlings of the lines OE1, OE25, and OE29 compared with the wild-type Col-0 grown in Pi-replete medium (calibrator Col-0; normalized to *UBQ10*). (b) Immunological detection of fusion protein AtPECP1-cMyc using anti-cMyc monoclonal antibody. (c)–(f) Levels of head group metabolites in the leaves (black bars) and the roots (grey bars) of overexpression lines OE1, OE25, and OE29 compared with wild-type Col-0 organs determined using HILIC-MS/MS; phosphoethanolamine (c), ethanolamine (d), phosphocholine (e), and choline (f). The key as seen in (d) applies to (c)–(f). Samples were taken from plants grown hydroponically on +Pi medium (0.5 mM) under long-day conditions for 35 d. Two-way ANOVA was used to evaluate the differences between organs and genotypes. Values represent the mean ±SD (*n*=5 biological replicates). Different letters indicate means that differ significantly (*P*<0.05).

In order to analyse effects on plant metabolism under Pi-replete conditions, we characterized three overexpression lines (*PromS35:AtPECP1-cMyc*), namely OE1, OE25, and OE29. These lines produced 100- to 1000-fold more *PECP1* transcripts than Col-0 seedlings (Fig. 7a). Using a cMyc antibody, we verified protein production in transgenic plants (Fig. 7b). PECP activity in overexpression lines ranged from 23 nmol to 30 nmol Pi mg^−1^ protein, which was ~50–100% greater than measured in whole Col-0 seedlings (15 nmol Pi mg^−1^ protein). We further used the selected lines to analyse organ-dependent transcript accumulation in 35-day-old adult plants, and found that the expression in roots exceeded the expression in leaves remarkably. Phenotypic changes were not observed in adult P-replete or P-starved overexpression lines compared with Col-0 plants (Supplementary Fig. S4). Free Pi concentrations of OE lines were similar to those in the Col-0 plants and vector control plants (Supplementary Fig. S3).

We analysed the levels of head group metabolites in leaves and roots of 35-day-old plants. The EA level was significantly elevated in leaves of OE plants in comparison with Col-0 plants (Fig. 7d, black bars), whereas the levels of the other head group metabolites were not altered (Fig. 7c, e, f, black bars). In roots of OE lines, substantial changes of steady-state levels of all head group metabolites occurred (Fig. 7c–f, grey bars). In line with our working hypothesis, PEA levels in overexpression lines were significantly reduced by ~50% when compared with Col-0 plants (Fig. 7c), while EA levels increased 2-fold to 5-fold. Accordingly, PCho levels were also significantly reduced in OE lines as compared with Col-0 plants, which may result from the lower PEA level since PEA is the main substrate for PCho synthesis. Interestingly, roots of OE mutant lines contained more Cho than wild-type roots (~2-fold). Similarly, inflorescence stems of PECP1-overexpressing plants contained significantly elevated EA and Cho amounts (Table 2). Concentrations of head group metabolites in control plants carrying an empty T-DNA cassette (pGWB vector control) were similar to those of Col-0 plants (Table 2; Supplementary Table S3).

**Table 2.** Ectopic *AtPECP1* expression results in accumulation of head group metabolites in inflorescence stems

Line	Ethanolamine (µmol g^−1^ DW)	Choline (µmol g^−1^ DW)
Col-0	2.48 ± 0.53 a	12.54 ± 1.54 a
OE1	5.71 ± 2.03 b	22.66 ± 1.44 b
OE25	5.78 ± 2.31 b	22.60 ± 8.20 b
OE29	18.32 ± 5.95 c	35.90 ± 15.68 c
VC	2.83 ± 0.22 a	12.55 ± 1.32 a

Comparison of contents in wild-type Col-0 plants, three overexpression lines (*PromS35::AtPECP1-cMyc*), and a plant line which carries an empty T-DNA cassette (pGWB vector control; VC). Plants were grown hydroponically on +Pi medium in long-day photoperiods for 35 d. One-way ANOVA was used to evaluate the differences between genotypes. Values shown represent the mean of five biological replicates ±SD. Different letters indicate means that differ significantly (*P*<0.05).

To investigate further the potential role of PECP1 in modulating the lipid composition, we analysed root samples from wild-type and OE plants lines, OE1, OE25, and OE29, grown in hydroponic culture and supplied with Pi-replete medium. Data on all main lipid classes comprising glycolipids (phospho- and galactolipids), DAG, and TAG, did not indicate that PECP1 impacts metabolic pathways of these lipid classes (Supplementary Table S4).

## Discussion

In this study, we investigated the enzymatic function and metabolic role of PECP1 from *A. thaliana*, a phosphatase of the HAD superfamily (May *et al.*, 2012). Having its strongest expression in roots of phosphate-starved plants (Fig. 2c), the *AtPECP1* gene is an important member of PSR genes in Arabidopsis. *AtPECP1* is co-expressed with PSR genes related to lipid metabolism (Lan *et al.*, 2015). The isolation of *Atpecp1* null mutants in combination with the knowledge on the organ-dependent shift of metabolite levels of phosphorylated and free head groups during Pi starvation (Fig. 3) allowed us to address the question of the biological role of AtPECP1.

The dramatic collapse of the PCho pool in roots of Pi-deficient plants (Fig. 3c), but also the moderate decrease in other plant parts, demonstrates that PCho represents an important nutritional phosphate source and contributes to the intracellular recycling of Pi (Plaxton and Carswell, 1999). Although having a much smaller pool size, PEA was also targeted by Pi starvation, again most strongly in roots (Fig. 3a). Importantly, the decline of the PCho content was detected both in all Pi-starved mutant *pecp1* lines in which AtPECP1 activity is absent, and in the wild type (Fig. 5c). This result argued against the involvement of AtPECP1 in PCho hydrolysis. In addition, our results supported the hypothesis that AtPECP1 hydrolyses solely PEA in Arabidopsis: first, Pi-starved *pecp1-1* plants exhibited reduced PEA-hydrolysing enzyme activity than wild-type plants, while PCho hydrolysis was similar (Fig. 4c). Secondly, all *pecp1* lines had significantly greater PEA levels but less EA than wild-type plants during Pi deprivation, which can be interpreted as the direct effect of the absence of a Pi starvation-inducible phosphoethanolamine phosphatase activity (Fig. 5a, b). In agreement with these results, there was no decrease of dephosphorylation activity of PCho in *pecp1* mutants, whereas PEA-dephosphorylating activity was strongly decreased (Fig. 4c). Thirdly, the ectopic expression of *AtPECP1* resulted in the accumulation of greater EA levels but smaller PEA amounts in the roots of the lines OE1, OE25, and OE29 under Pi-sufficient conditions (Fig. 7c, d), which was in accordance with the presence of additional phosphoethanolamine phosphatase activity.

**Fig. 8.**
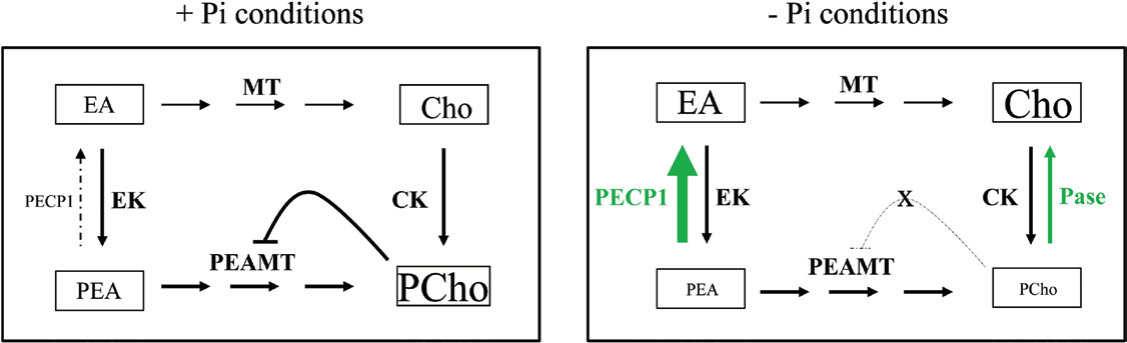
Model explaining the proposed function of the Pi starvation-activated PECP1 in the ethanolamine metabolism of Arabidopsis roots (font sizes represent relative metabolite levels but are not to scale). (a) During Pi-replete conditions, PCho synthesis by PEAMT is feedback regulated by the product PCho (Tabuchi *et al.*, 2006) which has a high level in roots (Alatorre-Cobos *et al.*, 2012). *PECP1* is barely expressed and the enzyme has no obvious function. (b) Pi starvation results in hydrolysis of the PCho pool by uncharacterized Pi starvation-induced phosphatases (Pases) as revealed by this study. A low PCho level would lead to enhanced PCho production through derepression of PEAMT translation and activity (Eastmond *et al.*, 2010; Craddock *et al.*, 2015). To prevent energy-consuming, superfluous PCho synthesis, Pi starvation-induced PECP1 reduces the PEA pool, the substrate of PEAMT.

It is well documented that phospholipid synthesis ceases when phosphate is only scarcely available, and both PCho and phospholipids are exploited as sources of nutritional Pi (Gout *et al.*, 1990; Plaxton and Carswell, 1999; Misson *et al.*, 2005; Plaxton and Tran, 2011; Nakamura, 2013; Siebers *et al.*, 2015; Pant *et al.*, 2015*a*). During Pi starvation, when PSR phospholipases C such as NPC4/NPC5 produce surplus PEA, transcriptional activation of *AtPECP1* may function to balance the PEA pool which is shared by PtdEA and PCho/PtdCho biosynthetic pathways. In addition, reducing the PEA level offers an important feedback control to curtail the production of PCho when this product is not required. We assume that the regulatory function of AtPECP1 is to down-regulate the *de novo* production of PCho through the methylation pathway when plants suffer phosphate deficiency. PCho synthesis is an energy-consuming process since each molecule of PCho produced requires seven molecules of ATP for EA phosphorylation, *S*-adenosyl-l-methionine production, and *S*-adenosyl-l-homocysteine recycling (Moffatt and Weretilnyk, 2001). The key enzyme in the methylation pathway is PEAMT1. Transcriptional repression of the *PEAMT1* gene in response to Pi starvation which would result in down-regulation of PCho synthesis was not found. While the study of Misson *et al.* (2005) reported an induction of *PEAMT1* (At3g18000) expression in leaves, Morcuende *et al.* (2007) could not confirm this result. Our own qRT-PCR data corroborate the view that the *PEAMT1* gene is not transcriptionally regulated by Pi starvation (data not shown). In contrast, PEAMT1 is subject to regulation on the translational and biochemical level (Fig. 8). The 5'-untranslated region of *PEAMT1* contains an upstream ORF (uORF) which enables translational repression of PEAMT1 by PCho without significantly affecting its steady-state transcript abundance (Tabuchi *et al.*, 2006; Eastmond *et al.*, 2010; Alatorre-Cobos *et al.*, 2012; Craddock *et al.*, 2015). This behaviour is consistent with previous reports on the inhibition of PEAMTs from different plant species by PCho (BeGora *et al.*, 2010). It seems very likely that the very low PCho level in Pi-deprived roots (Figs 3c, 5c) results in derepression of PEAMT1 translation and activation of PCho synthesis (Fig. 8). Taken together, the implementation of a regulatory step such as *AtPECP1* transcriptional activation is an elegant strategy when regulatory features working under Pi-replete conditions are no longer effective. Consistently, *AtPECP1* was barely expressed and had no obvious function under Pi-replete growth conditions. Since neither the loss of AtPECP1 nor its overexpression impacted the free Pi concentration, a regulatory function of AtPECP1 in head group metabolism seems more likely than a role in remobilization of phosphate. Recent studies showed that previously uncharacterized phosphatases of the HAD superfamily function in the removal of phosphorylated intermediates that accumulate when pathways are stalled (Allen and Dunaway-Mariano, 2009), in this case due to lipid remodelling induced by the lack of phosphate, and that may lead to enzyme repression by the accumulating head groups.

Intriguingly, the 35S promoter-driven ectopic expression of *AtPECP1* in Pi-replete plants resulted in modification of the head group metabolism apart from PEA and EA. PEA is an important precursor for PCho synthesis and, therefore, the observed reduction in PEA level most probably hampers the synthesis of PCho and results in a significantly lower PCho content in roots of *AtPECP1*-overexpressing plants (Fig. 7e). The opposite could happen with regard to the elevated EA level since the accumulation of EA enables increased flux into Cho. Arabidopsis mutants deficient in *PEAMT1* gene expression have a severe reduction of up to 64-fold in Cho content, demonstrating that PEAMT enzymes control the metabolic flux to Cho (Mou *et al.*, 2002). Consistently, tobacco plants overexpressing *PEAMT* produce 5-fold more PCho and 50-fold more free Cho (McNeil *et al.*, 2001). These findings suggest that PEAMT activity was able to modulate the Cho synthesis rate in *AtPECP1*-overexpressing plants which grew under Pi-sufficient conditions without metabolic restrictions. An interesting point is also that Cho accumulated in roots of OE plants and was not immediately phosphorylated by choline kinases. In certain species Cho serves as precursor for the synthesis of osmoprotectants such as glycine betaine (GB) and choline-*O*-sulphate. It might be worth proving the usefulness of PECPs in attempts to modulate Cho synthesis and stress tolerance (Rontein *et al.*, 2002; Reguera *et al.*, 2012).

In both the loss-of-function *Atpecp1* lines and the ectopic AtPECP1-overexpressing lines, the impact on head group metabolite levels is greatest in Arabidopsis roots. It indicates that PCho and PEA metabolism in roots is more sensitive to enzymatic changes than the phospholipid metabolism of shoots (Hocquellet *et al.*, 2005). Our data are in line with results of recent studies suggesting that PCho biosynthesis takes place preferentially in roots and is tightly controlled by *PEAMT1* gene expression in roots and uORF-mediated translational regulation of PEAMT1 (Cruz-Ramírez *et al.*, 2004; Alatorre-Cobos *et al.*, 2012; Craddock *et al.*, 2015). Experiments using radiolabelled Cho revealed that (i) the root is a site of synthesis of PCho and PEA for phospholipid synthesis in tomato leaves and (ii) metabolites are transported in the xylem (Martin and Tolbert, 1983). Further experiments including organ-specific AtPECP1 overexpression lines are necessary to clarify whether the observed increase of EA and Cho levels in inflorescence stems of OE lines (Table 2) results from modified metabolism and/or from root export. Instead of using a synthetic medium, it would also be advisable to grow plant lines in different soils, and in the presence of naturally occurring phosphate concentrations which either sustain maximum growth or are limiting. We could thus estimate the impact of PECP1 on head group metabolism in both environments.

It remains an open question what enzymes function to dephosphorylate PCho during Pi starvation. The results showed that there were uncharacterized PSI enzyme activities in soluble cell extracts which were able to hydrolyse PCho (Fig. 4c). A universal plant response to Pi deprivation is the up-regulation of a diverse array of phosphatases which catalyse Pi hydrolysis from a broad and overlapping range of phosphate-monoesters (Plaxton and Tran, 2011). A cell-wall associated PCho-hydrolysing enzyme activity has been demonstrated in sycamore cell cultures (Gout *et al.*, 1990). Further work is necessary to discover the enzymes that contribute to PCho hydrolysis, together with their cellular localization and spatial distribution.

## Supplementary data

Supplementary data are available at *JXB* online.

Fig. S1. *AtPECP1* expression in soil-grown plants and PECP1 subcellular localization.

Fig. S2. PCR genotyping of the T-DNA insertions in the *AtPECP1* gene.

Fig. S3. Free phosphate concentrations in seedlings and plants.

Fig. S4. Phenotypes of phosphate-replete and phosphate-starved AtPECP1 overexpression lines.

Table S1. Primer sequences.

Table S2. Multiple reaction monitoring transitions (HILIC-MS/MS).

Table S3. Comparison of head group metabolite contents between Col-0 and vector control plants.

Table S4. Comparison of root lipid composition between Col-0 and AtPECP1 overexpression lines.

Supplementary Figures and Tables
