# Supplementary material for: Pi starvation-dependent regulation of ethanolamine metabolism by phosphoethanolamine phosphatase PECP1 in Arabidopsis roots
Source: J Exp Bot. 2017 Dec 22;69(3):467–81. doi: 10.1093/jxb/erx408 (PMC5853852; doi:10.1093/jxb/erx408)
Supplement: Supplementary Figures and Tables [file erx408_suppl_supplementary_figures_and_tables.pdf]

Supplementary data

Supplementary Figure S1

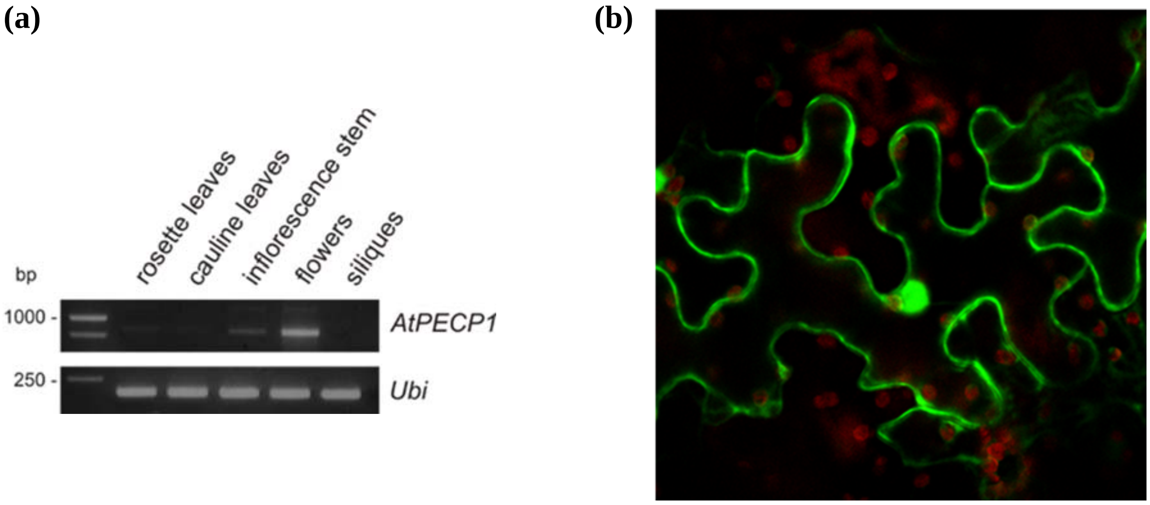

Figure S1: *AtPECP1* expression and subcellular localization of AtPECP1

(a) Semi-quantitative PCR analysis of different organs of soil-grown, adult Arabidopsis plants (Loading control *UBQ10*)

(b) Subcellular localization of AtPECP1 in *Nicotiana benthamiana* leaves using confocal laser scanning microscopy. The *Prom35S:PECP1-eYFP* construct was delivered via *Agrobacterium*-mediated transformation.

Supplementary Figure S2

(a)

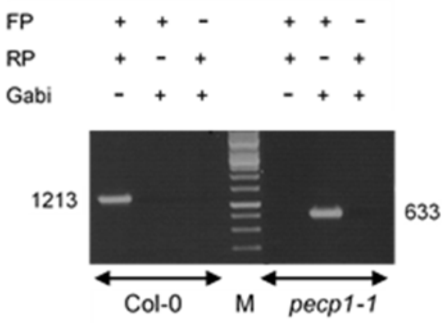

(b)

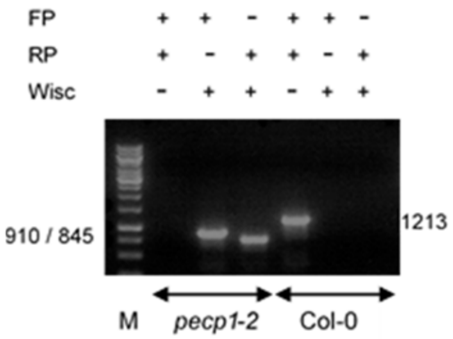

(c)

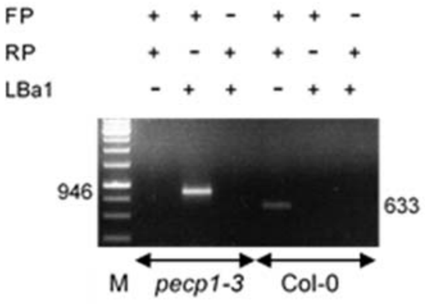

Figure S2: PCR genotyping of the T-DNA insertions in the *AtPECP1* gene (At1g17710). Forward primer (FP) and reverse primer (RP) refer to gene-specific primers. Primers designated Gabi, Wisc, or LBa1 are T-DNA-specific primers used for the respective line. Sizes of the PCR products (bp) using wild-type DNA (Col-0) as template or sizes of T-DNA-dependent PCR products (bp) or are written next to the bands, respectively. Primers used for genotyping are listed in Supplementary Table S1 (Analysis of T-DNA insertion lines).

Supplementary Figure S3

(a)

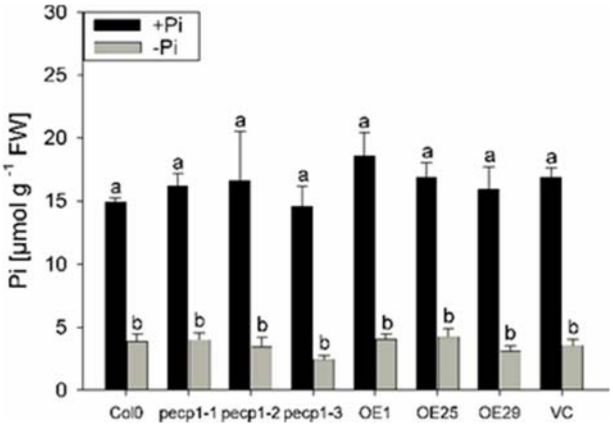

(b)

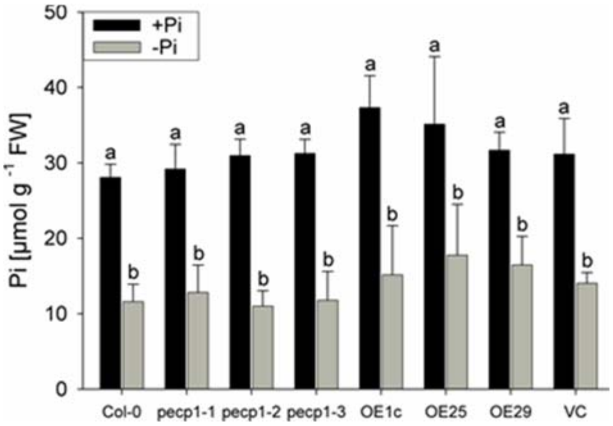

(c)

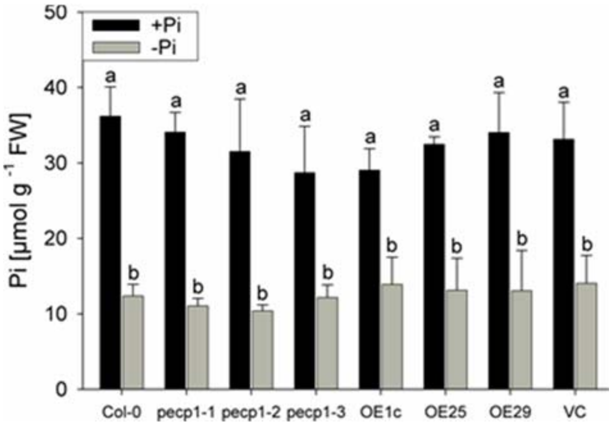

Figure S3: Measurement of inorganic phosphate concentrations in seedlings, roots and shoots of Col-0 wild-type plants, *AtPECP1* loss-of function plants (*pecp1* mutants), *AtPECP1* gain-of-function plants (OE lines), and control plants carrying an empty vector (VC)

(a) 14-day-old seedlings cultivated on agar plates containing 1 mM Pi (+Pi) or 20 µM Pi (-Pi)

(b) Roots of 28-day-old plants grown in hydroponics under P-replete conditions (+Pi, 0.5 mM Pi) or low-Pi conditions (-Pi; 10 µM Pi)

(c) Shoots of 28-day-old plants grown in hydroponics under P-replete conditions (+Pi, 0.5 mM Pi) or low-P conditions (-Pi; 10 µM Pi)

Two-way ANOVA was used to evaluate the differences between genotypes and treatments. Values shown represent the mean ± SD (n = 5 biological replicates). Different letters indicate means that differ significantly ($P<0.05$).

Supplementary Figure S4

(a)

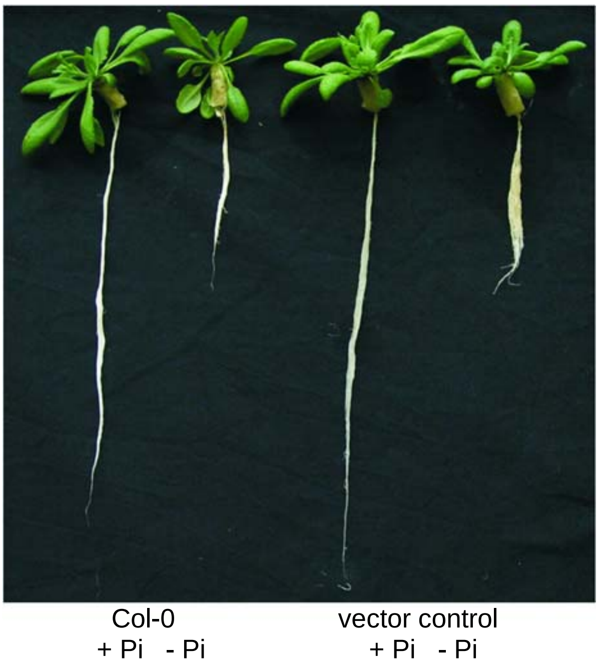

(b)

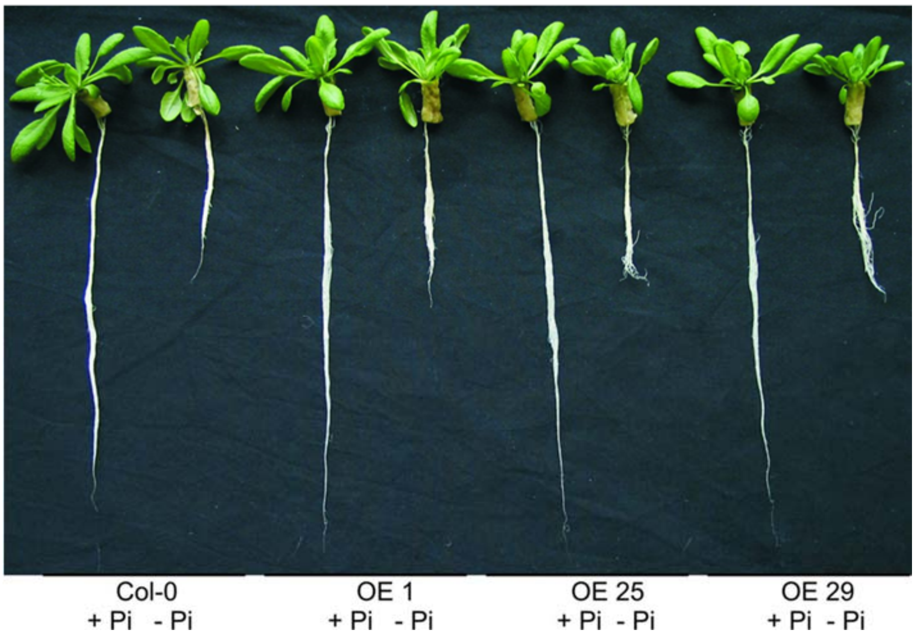

Figure S4: Phenotypes of AtPECP1 overexpressor plants

(a) Comparison of Col-0 plants and vector control plants, grown in hydroponics under Pi-replete (+Pi; 0.5 mM Pi) or low-P conditions (-Pi; 10 µM) for 28 days.

(b) Comparison of Col-0 plants and overexpressor plants (lines OE1, OE25, OE 29), grown in hydroponics under Pi-replete (+Pi; 0.5 mM Pi) or low-P conditions (-Pi; 10 µM) for 28 days.

Supplementary Table S1

Sequences of primers used in this study

(* Marked primers are placed 3' of the respective T-DNA insertion)

Method	Primer name	Sequence [5' ⟶ 3']
sqRT-PCR	sq-PECP1_f<br>sq-PECP1_r<br>sq-Ubi_f<br>sq-Ubi_r	ATG GCT AAG AAT AAC AAC ATC GTG<br>TCA CTT GAC CAA ATT TAA AGG AAC<br>TGG ACG TAC TTT GGC CGA CT<br>AGA GGC TGT ACA TGG CCC CA
qRT-PCR<br>(all plant lines except *pecp1-1*)	qRT-PECP1_f *<br>qRT-PECP1_r *<br>qRT-Ubi_f<br>qRT-Ubi_r	CGT TAG AGA TTG GAC CGA TGG A<br>CAA TCC CAA CAG ATA TCT TGC AGT<br>TGG ACG TAC TTT GGC CGA CT<br>AGA GGC TGT ACA TGG CCC CA
qRT-PCR<br>(*pecp1-1*)	qRT-*pecp1-1*_f *<br>qRT-*pecp1-1*_r *	CGA GCT GAG AAT AGT GAG CGA C<br>TCA CTT GAC CAA ATT TAA AGG AAC
Analysis of T-DNA insertion lines<br>(gDNA / PCR)	FP (*pecp1-1; 1-2*)<br>RP (*pecp1-1; 1-2*)<br>FP (*pecp1-3*)<br>RP (*pecp1-3*)<br>Gabi<br>Wisc<br>LBa1	ATG GCT AAG AAT AAC AAC ATC GTG<br>TCA CTT GAC CAA ATT TAA AGG AAC<br>CGA GCT GAG AAT AGT GAG CGA C<br>CTT GTC ATT CTC CTC TCC TTC CTC TG<br>CGC CTA TAA ATA CGA CGG ATC<br>CAT AAT AAT GTG TGA GTA GTT CCC<br>TGG TTC ACG TAG TGG GCC ATC G
Cloning OE / eYFP	entr-PECP1-f<br>entr-PECP1-r	CAC CAT GGC TAA GAA TAA CAA CAT CG<br>GCA CTT GAC CAA ATT TAA AGG

Supplementary Table S2

Multiple reaction monitoring (MRM) transitions for the HILIC-MS/MS analysed phospholipid head groups indicating the selected masses of the precursor ion (Q1) and the fragment ion (Q3) as well as the declustering potential (DP), the collision energy (CE) and the cell exit potential (CXP) used for the fragmentation.

ID	Q1 Mass [Da]	Q3Mass [Da]	Dwell time [msec]	DP [V]	CE [V]	CXP [V]
PEA	139,901	78,8	20	-45	-18	-9
EA	62,024	44	20	6	13	10
PCho	184,036	125	20	106	25	8
Cho	104,056	58	20	56	21	8
PEA-d4	143,901	78,8	20	-45	-18	-9
EA-d4	66,024	48	20	6	13	10
PCho-d9	193,036	125	20	106	23	10
Cho-d9	113,17	66	20	56	25	8

Supplementary Table S3

Comparison of head-group metabolite contents between P-replete Col-0 plants and P-replete vector control plants. Two-way ANOVA was used to evaluate the differences between genotypes and organs. Values shown represent the mean of three biological replicates ± SD. Different letters indicate means that differ significantly (P<0.05).

	Roots [µmol g^{-1} DW]		Leaves [µmol g^{-1} DW]	
metabolite	Col-0	vector control	Col-0	vector control
phosphoethanolamine	2.86 ± 0.87 [a]	2.39 ± 0.27 [a]	1.02 ± 0.20 [b]	0.47 ± 0.06 [b]
phsophocholine	49.7 ± 5.04 [a]	52.92 ± 1.69 [a]	9.23 ± 1.22 [b]	6.81 ± 0.33 [b]
ethanolamine	2.81 ± 0.91 [a]	2.56 ± 0.23 [a]	2.47 ± 0.52 [b]	2.41 ± 0.99 [b]
choline	18.03 ± 1.52 [a]	22.18 ± 2.36 [a]	4.57 ± 0.84 [b]	4.28 ± 1.71 [b]

Supplementary Table S4

Comparison of root lipid composition between wild type plants (Col-0) and AtPECP1 overexpressing lines OE1, OE25, and OE29 (*Prom35S:AtPECP1-cMyc*) grown in Pi-replete medium for 35 days. Data shown are relative and represent normalized intensity units.

One-way ANOVA was used to evaluate the differences between genotypes. Values are means ± SD (n = 3 biological replicates with 3 plants each). Different letters indicate means that differ significantly (P<0.05).

	Col-0	OE1	OE25	OE29
PtdEA	234.41 ± 6.39 [a]	219.96 ± 6.95 [a]	229.20 ± 7.82 [a]	235.02 ± 2.81 [a]
PtdCho	697.05 ± 12.43 [a]	634.69 ± 36.26 [a]	655.26 ± 45.01 [a]	688.87 ± 9.61 [a]
SQDG	1.87 ± 0.09 [a]	1.87 ± 0.10 [a]	1.75 ± 0.04 [a]	1.72 ± 0.08 [a]
MGDG	24.24 ± 2.31 [a]	20.14 ± 7.11 [a]	24.50 ± 0.46 [a]	22.96 ± 0.81 [a]
DGDG	9.71 ± 0.74 [a]	9.67 ± 0.36 [a]	9.30 ± 0.30 [a]	9.83 ± 0.09 [a]
DAG	10.44 ± 0.15 [a]	10.78 ± 0.78 [a]	10.31 ± 0.29 [a]	10.14 ± 0.67 [a]
TAG	45.06 ± 0.37 [a]	44.07 ± 3.61 [a]	44.03 ± 0.67 [a]	49.53 ± 3.94 [a]
LysoPE	1.23 ± 0.01 [a]	1.36 ± 0.15 [a]	1.32 ± 0.03 [a]	1.48 ± 0.10 [a]
LysoPC	1.60 ± 0.01 [a]	1.73 ± 0.17 [a]	1.75 ± 0.03 [a]	1.89 ± 0.15 [a]
